# The Impact of Customer Experience and Customer Engagement on Behavioral Intentions: Does Competitive Choices Matters?

**DOI:** 10.3389/fpsyg.2022.864841

**Published:** 2022-05-25

**Authors:** Bilal Ahmed, Shagufta Zada, Liang Zhang, Shehla Najib Sidiki, Nicolás Contreras-Barraza, Alejandro Vega-Muñoz, Guido Salazar-Sepúlveda

**Affiliations:** ^1^School of Business, Qingdao University, Qingdao, China; ^2^Department of Business Administration, ILMA University, Karachi, Pakistan; ^3^Faculty of Management Sciences, Shaheed Zulfikar Ali Bhutto Institute of Science and Technology (SZABIST) University, Karachi, Pakistan; ^4^Facultad de Economía y Negocios, Universidad Andres Bello, Viña del Mar, Chile; ^5^Public Policy Observatory, Universidad Autónoma de Chile, Santiago, Chile; ^6^Departamento de Ingeniería Industrial, Facultad de Ingeniería, Universidad Católica de la Santísima Concepción, Concepción, Chile

**Keywords:** customer cognitive engagement, affective engagement, behavioral engagement, customer experience, customer identification, behavioral Intention, competitive choices

## Abstract

The study aims to analyze behavioral intentions influenced by customer engagement, experience, and identification moderated by competitive choices in the granite sector of Pakistan. The study has been carried out through primary data analysis of cross-sectional approach in the transition to a sustainable economy. In total, 400 questionnaires were distributed, for which only 216 were filled and usable with a response rate of 54%—collected data from the production managers and units. In contrast, missed mine holders and labor analyzed the data in SPSS and AMOS to run various tests, i.e., reliability, correlation analysis, regression, moderation regression, and confirmatory factor analysis. The study findings indicate a positive and significant relationship and effect among the variables. The reviews might contain some biases. Therefore, this study recommended adopting a probability sampling technique for future studies. The study results in a positive manner indicating customer service involvement as a significant factor in behavioral intention despite competitive options.

## Introduction

The rising trend of globalization with an intense speed and the new paradigms of sustainability and sustainable economies have not only brought the world market to a single place. However, it has also brought fierce competition (Le et al., 2019). Cutthroat competition, economic crises, and new technologies have brought opportunities and threats as well (Hultman et al., 2015; Hollebeek et al., 2019). Granite is an ancient stone loved by the Romans for construction, paving, and erecting columns (Wilson, 1988; Poggi and Lazzarini, 2005; Careddu et al., 2017). The granite stone quarried at that time was used for pavement structure (especially in Genoa and Rome), historic sites (i.e., the war memorial erected at Ismailia in Egypt in 1930, the monument to Bartolomeu de Gusmão in Santos in Brazil), and for buildings in Italy and around the world (Careddu et al., 2017).

Granite is known as “King of Stones” because of its inherent characteristics, such as extra fine mirror polish, scratch-free glossy surface, and durability (Sudarsan, 2017). Granite produced in Pakistan has become the most sought after and extensively used stone material in building constructions and massive structural works throughout the world and is well known in the international market not only for its elegance and esthetic quality but also for its durability. This study analyses the experience and engagement of customers in granite stones. Also, it analyses the factors that influence them to buy the granite stones with expertise in the study area (Kumar, 2010).

Since the start of the twenty-first century, different analysts have recommended experience as the capacity to increase the value of a brand (Vargo and Lusch, 2014). This element permits organizations to foster a differentiated competitive edge (Ali et al., 2018) based on a creative offering presented in a one of a kind (Blut et al., 2014) and in a specified manner (Bilgihan et al., 2016). This new customer-centricity vision (Kohli et al., 2019) infers a piece of extraordinary information on their behavior and needs (De Mooij, 2019).

Experiences are “higher mental processes, for example, insight, memory, language, critical thinking, and unique reasoning” (APA, 2016). They have been examined comparable to customer experience using the accomplishment of objectives and disconfirmation of earlier assumptions. The point of view is established in the supposition that buyers are goal-directed in their behavior. For example, customers buy vitamins for health; they do grocery shopping to enjoy food. Customers consciously or unconsciously set up goals in specific contexts and use consumption as an instrument to attain them (Bagozzi and Dholakia, 1999). The marketing literature describes goal-directed behavior as a cognitive process; attainment of goals results from this process (Baumgartner et al., 2008). Thus, achievement of goals constitutes one part of customer experience, and reflecting on goal attainment is crucial to evaluate the cognitive element of customer experience (Novak et al., 2003).

The physical environment, or “services cape” (Bitner, 1992), refers to the manufactured, firm controllable surroundings within which service experience occurs. Offline services capes comprise elements such as sense, touching, shades, texture, and layout Lam, 2001). Online services cape considerations focus on website features, such as consumer-friendly shopping interfaces (Griffith, 2005) and design cues such as uncluttered screens and brief presentations (Rose et al., 2012). The customers’ interactions with services capes have been shown to influence their experiences. For example, within offline settings, impacts are observed on satisfaction, facility image perceptions, word of mouth behaviors and intentions to purchase (Wakefield and Blodgett, 1996; Reimer and Kuehn, 2005). Ambient conditions comprise background environmental stimuli (Grayson and McNeill, 2009), including visual (e.g., lighting, colors, and shapes) (Dijkstra et al., 2008), esthetic cleanliness, olfactory (e.g., scent and air quality) (Mattila and Wirtz, 2001), temperature (Reimer and Kuehn, 2005), and auditory (e.g., music and noise) (Garlin and Owen, 2006; Oakes and North, 2008) elements. Spatial layout refers to the way items such as equipment and furniture are arranged, their size and shape, and the space between them (Edvardsson et al., 2010) and lesser visual elements such as comfort, accessibility (Wakefield and Blodgett, 1996), and functionality (Ng, 2003). In offline and online scenarios, spatial layout and functionality considerations relate to design (Hultén, 2012) with outcomes linked to customer perceptions, and behavioral responses such as unplanned purchase behavior (Inman et al., 2009), and increased sales and willingness to spend (Fiore et al., 2000). Rose et al. (2012) highlighted the relationship between ease of use (site navigation, search, and functionality) and cognitive perceptions of control in an online environment. The self-service technology research stream (Curran and Meuter, 2005) further emphasizes the importance of functionality within services capes where customers need to perform the service. Signs, symbols, and artifacts are used as communication tools to stimulate more abstract customer meaning-making (Thompson and Arsel, 2004; Rosenbaum and Massiah, 2011).

Notwithstanding these studies, the customers are the exporters and importers who proactively create business associations to adapt adequately to the countries’ sustainability requirements (Jaakkola and Aarikka-Stenroos, 2019). Customers mostly enter such business relationships with those sellers who admire customer value through such a relationship. Therefore, value creation becomes a continuous process of business success. The communication between seller and customer is complex, which comprises investment in the success of the business relationship. In contrast, the risk of loss is also emerging. Customers mainly collaborate with agents who deal in various markets internationally and possess multiple language skills, which create a passage of interaction for the seller and customer.

Academicians consider the granite industry to improve seller and customer relationships (Leonidou and Hultman, 2019). In this scenario, certain technological advancements and labor expertise that meet customers’ requirements are crucial (Anandaraman, 2017). As granite increases globally and purchasing, the industry’s language-perfect know-how remains limited on the national borders (Kumar et al., 2019). The literacy rate remains minimal, a significant obstruction for international buyers or customers to meet their demands. Satisfactorily increases the gap for educated entrepreneurs to enter the stone industry; such factors indicate a less or almost no seller and customer relationship, which has motivated the author to develop a deep understanding of customer experience. This study discusses understanding customer behavioral intention, the importance of a customer’s engagement, customer experience, and industry information (Choi and Kim, 2013). Customer experience is conceptually distinct from service quality judgment (Schmitt, 1999). In the study by Verhoef and Lemon (2013), customer experience is considered a crucial component. In the context of service, customer experience is an essential part of the service and goods industry. A customer relationship is an integral part of the goods and services industry. Nevertheless, besides such importance, business venture’s success and customer experience have not been discovered yet (Arshad and Habib, 2018; Altinay et al., 2019; Lazard et al., 2020).

According to the study by More (2019), the global natural stone market size was valued at $35,120.1 million in 2018 and is projected to reach $48,068.4 million by 2026. The consumption of natural stones increased three times in the United States from 1994 to 2003 and is expected to reach billions of US dollars by 2025. After China, the United States was the second most crucial natural stone buyer (Bai et al., 2020). The annual export of 2018 for the stone was $445.4 million (Workman, 2019). The need for granite rather than marble or other natural stone increases with time due to its hardness and visibility (Biró et al., 2019). European countries, Asia (Pakistan, China, and Malaysia), and Africa possess important industrial sectors to alter dimensional stones (Brocx and Semeniuk, 2019). Remains and fossils of ancient quarries still exist in the vicinity of Pakistan and worldwide (Careddu et al., 2017). Granite is used globally as an essential building material, mainly for cladding and flooring (Schmitt, 1999). Granite and other dimensional stones have been mined previously by non-industrial local laborers on a small scale (Ahmad and Khan, 2019). Granite blocks have been exported on a lower scale, mainly to China since 2007 (Recorder, 2019). A considerable investment in the granite sector started in manufacturing plants through mining and factories with increased production and reserves of 1,000 billion tons (Hussain, 2017).

The greater incorporation of customer engagement into marketing literature will encourage researchers to consider some aspects that need greater research attention. First, businesses spend billions of dollars on potential or future customers to extend the value of their brand. For instance, Microsoft and Apple provide computers to schools, creating goodwill and positive brand reinforcement (Morgan et al., 2021). Clinique organizes makeup workshops, called “Attracted to Color,” twice a year to enable anyone who wishes to have an opportunity for a one-on-one consultation with its makeup experts. These efforts aim to establish customers’ engagement with the brand, whether or not a purchase is an immediate prospect. Second, many current programs are not purchase focused and instead focus on achieving engagement with all the interested parties. For example, American Express, through the Members Project, urges card members “to dream up, and ultimately unite behind, one incredible idea. American Express will bring it to life with up to $5 million” (Shiri and Rathi, 2013). Third, existing and potential customers often interact among themselves. This interaction strongly influences their consumption decisions, given that other customers may be more influential than company advertising. For example, potential customers often read online reviews from other customers and product reviews before buying a product. Fourth, CE within the RM research will allow for consideration of the opportunities provided by organizations for interactions among customers and prospects focused on helping them and sharing experiences to solve one another’s problems. Such as the baby birth or cancer seminars in many hospitals that bring patients together in similar life situations (Beatty, 2019).

Several studies focused on Pakistan being one of the stone-enriched countries in the world and have a great deal of potential for prosperity, economic development, and exports (Omair et al., 2015; Rashid et al., 2020). Pakistan has a reserved deposit of 600,000 square kilometers of metallic and non-metallic minerals and ores (Rather, 2020). Dimensional stone is regarded as an emerging trade in the economy of Pakistan. Pakistan specifically masters in the business of black galaxy granite in terms of specifications such as hardness and hotness. The texture of the black granite varies globally among international producers.

Although past studies examine the effects of customer engagement on the customer experience, identification, and indirect impact of engagement on behavioral intentions through identification and experience (Arrfat, 2020; Safitri et al., 2020). This study traces out that no previous research has been done between customer engagement, customer experience, and information on behavioral intention with the moderating role of competitive choices. Second, to previous results, the study has proposed to imply the moderating impact of competitive decisions on the association to understand the association further and clearly among constructs. With a practical significance, this study uses the theoretical model of the association of customer engagement with the customer experience and identification through other choices available. Third, the first part of the study covers the introductory section. The literature has been covered in the second section; methodology in the third; correlation, regression, and SEM (structural equation modeling) analyses. Furthermore, in the fourth section and discussion, conclusion, and limitations of the study in the last area of the study. The introduction triggers the research questions presented later:

a. Does customer involvement in the manufacturing sector impacts behavioral intentions?

b. Does competitive choices or options matter under customer involvement?

## Literature Review

### Customer Engagement and Behavioral Intention

Can recognize the growth of global trade activities of a firm through the customer engagement process. In today’s cutthroat competition, firms that engage with their customers jointly can quickly increase firm performance in various manners and brand recognition (Gärtner, 2014). Previous studies have discovered customer engagement as a critical success element for almost every business entity (Verhoef et al., 2010; Kumar and Pansari, 2015). In this case, commitment from customers toward businesses do not remain limited to purchases but also includes non-tangible customer activities (Kumar and Reinartz, 2016). All these commitments from customers toward the industry have been obliged to interpret customer engagement (Kumar et al., 2010; Van Doorn et al., 2010). However, the concept of customer engagement has been discussed in the various fields of psychology (as task engagement), and marketing (customer engagement) (Verhoef et al., 2010; So et al., 2014; Ahn and Back, 2018; Hollebeek et al., 2019). The dimensions of customer engagement had been in vast discussion topic for the researchers. Perceptively, several researchers have suggested customer engagement to comprise intra and extra role customer emotions, cognitions, and behavioral (Islam et al., 2019; Kumar et al., 2019), while a few researchers limit the dimensions toward extra role exclusively (assisting behaviors) (Van Doorn et al., 2010). This study employs the previous paradigm that provides the most effective, comprehensive insight into customer engagement (Hollebeek et al., 2014, 2019; Harrigan et al., 2018a). Furthermore, given the communicative conceptual origin (Brodie et al., 2011), customer engagement has been considered from the perspective of relational marketing (Rather, 2019) and SD logic aspect (Hollebeek et al., 2019). These researchers believe customer engagement is customer initiative support in their communication (Kumar et al., 2019).

Customer engagement is considered a psychological state under customer interaction experiences with an object, brand, or person (Brodie et al., 2011; Zada et al., 2022a). In addition, Van Doorn et al. (2010) described customer engagement as actions that go ahead of capital investments and are generally defined as a customer’s moral expression that has any brand, firm, or person focus, above purchases, concluding motivational intentions. Apart from these different perspectives, had broadly elaborated customer engagement to incorporate affective, cognitive, and behavioral aspects, uncovering its multifaceted perspective (Hollebeek et al., 2014; Taheri et al., 2014; Harrigan et al., 2018a). Thus, the exception of customer engagement’s psychological viewpoints or behavioral aspects would almost certainly result from inadequate knowledge to appropriately examine the concept (Hollebeek et al., 2014; Ahn and Back, 2018). Subsequently, alone neither the psychological perspective nor behavioral aspect demonstrates customer engagement fully should consider both these aspects highly to understand the phenomenon of customer engagement (Ahn and Back, 2018; Hollebeek et al., 2019). Therefore, this study focuses on tri–dimensional customer engagement, i.e., affective, behavioral, and cognitive (Hollebeek et al., 2014; Ahn and Back, 2018). The cognitive aspect refers to the customer’s level of a firm, brand, or person (seller) thinking process and how to interactively understand a particular aspect, brand, firm, or person. The affective aspect is the customer’s aspect of a positive brand, firm, or relationship in a specific customer and seller interaction. At last, behavioral customer engagement is the amount of energy, time, and effort a customer spends on a brand, firm, or person in a specific customer and seller interaction (Hollebeek et al., 2014).

Customer affective engagement involves the customer’s interaction with the brand or a product. It simply is the number of times a customer uses or consumes a product, brand, service, or with a person; the level of experience, knowledge, and identification will increase and *vice versa*. A customer cognitive engagement refers to the customer’s level of expertise to understand a specific product, brand, person, or service in terms of performance, durability, or reliability. As the understanding relatively increases, a customer’s experience increases and *vice versa*. The behavioral aspect deals with time energy involving a product, firm, brand, person, or service that increases a customer’s level of experience and identification.

### Customer Experience and Behavioral Intention

Introduced the concept of experience in marketing, which became critical in understanding overall customer behavior through interaction experience (Hirschman and Holbrook, 1982; Lemon and Verhoef, 2016; Coudounaris and Sthapit, 2017). Experience marketing is a developing field of marketing philosophy (Tsaur et al., 2007; Song et al., 2015; Le et al., 2019) with significant implications in all aspects of marketing (Brun et al., 2017; Rather, 2019; Sharma and Nayak, 2019). Experiences are personal understandings that occur whenever a sense is encouraged in search of learning something new, primarily an outcome of direct participation in a virtual or real event (Schmitt, 1999). Experience is considered as an independent psychological condition experienced by customers (Tsaur et al., 2007). Generally, experiences are not self-created but achieved. Experiences are gained from the events environment whenever an individual is in contact.

Moreover, experience is described as a complicated, rising resource, i.e., no two experiences are mutual (Schmitt, 1999; Tsaur et al., 2007). The determinants of experience rose in the 1990s after economies changed (Pine and Gilmore, 1998). Economic activities aim not only for output but experience through interaction (Quan and Wang, 2004). Experiences limit or eradicate substitution or imitation based on a distinctive economic, competitive advantage (Arrfat, 2020). Accordingly, to the approaches of Hirschman and Holbrook (1982) and Lemon and Verhoef (2016) suggested customer experience management as an important development for marketing practitioners and authors. A few researchers have emphasized and urged the need for experiential marketing than conventional marketing practices (Schmitt, 1999). The proposed study argues that the customer desires interaction, product, brand, services, or persons. Marketing activities that melt their hearts arouse their minds, encourage, or motivate their senses and make them a part of their lives. Thus, customers desire interactions, marketing practices, and value propositions to enhance their experience.

Customer benefits from experiencing and using a product or service that involves their perspective of benefits received from the product or service and how the needs are fulfilled (Amenuvor et al., 2019). Conclusions from Zulfadli et al. (2019), the result of a product or service from a customer’s experience might be their conscious or unconscious evaluation of the product or service received. Consequently, after usage experiences will affect the customer’s intentions toward repurchasing or re-usage and recommend the product or service to others. Thus, behavioral intention can be affected positively or negatively by customer experience. Consequently, experience marketing is rapidly gaining importance among marketers and authors to create experiential relations with customers and consumers (Schmitt, 1999; Homburg et al., 2017; Le et al., 2019; Zhuang et al., 2021). It is a general psychological understanding that whenever one attains experience regarding any product, brand, or firm, the behavior toward that specific item also changes. After an experiential encounter, the customer might not feel the same as previously.

### Customer Identification and Behavioral Intention

The SIT or social identity theory is a significant conceptual base for customer identification in the marketing paradigm (Mael and Ashforth, 1992; Hultman et al., 2015; Rather, 2019). Based on SIT, customer identification indicates a customer’s mental condition of feeling, noticing, and examining their attachment with the product, brand, or offering (Rather and Hollebeek, 2019; Zada et al., 2021). Social identity theory suggests that customers can spend reasonable efforts to develop their social identity apart from personal identity (Bhattacharya and Sen, 2003; Rather et al., 2018). Such opinions are also favorable in SET or social exchange theory which concentrates on ones’ endeavors from social endeavor (Hollebeek, 2011), thus indicating significance among the concepts employed in this study. The author of this study applied identification in customer seller and brand relationship manner (Bhattacharya and Sen, 2003; Rather and Hollebeek, 2019). Author emphasized on customer’s social and personal identity which promote toward self-image. Customers involve in a comparable process to recognize brands or offerings that are compatible with their self-image (Escalas, 2004; Hultman et al., 2015). In this context, Sprott et al. (2009) suggested customer experience and customer identification are highly linked together, considers customer self-brand connection as an outcome of customer experience that arises from a customers’ specific communicative experiences (Hollebeek et al., 2014), defined customer identification as the relationship a customer creates with a brand and identity (Arrfat, 2020). Logically, this study supports affective, behavioral, and cognitive customer engagement influences customer identification. Similarly, the customer’s affective and cognitive customer engagement anticipates the customer’s relationship with the brand and identification of the offerings (Hollebeek et al., 2014). Relatively, Harrigan et al. (2018a) suggested that customers are involved in social networking, which increases identification. Precisely, this links a brand to a customer’s identity. SIT or social exchange theory acknowledges that customers should spend resources on value, i.e., compatibility, identification, and status (Hollebeek, 2011; Rather and Hollebeek, 2019). Customers exchange monetary, societal, intensive, cognitive, and physical re-serves with marketers (Hollebeek, 2011; Rather, 2019). For customer engagement to be equivalent assists both, i.e., customer and seller, explaining customer engagement as a social exchange process (Harrigan et al., 2018b; Rather, 2019).

Generally, whenever a customer or customer identifies the product or brand, it is alarmed by what is required in the senses. Identification can stimulate the senses and affect behavioral intention, whether positively or negatively. Mainly, customer identification results in a positive if the interaction with a specific brand, person, product, or service meets the desired quality and *vice versa*.

### Competitive Choices

The competitive choice is vital for both ends, i.e., customer and seller (buyer and consumer/customer) (McKenzie and Sansone, 2019). Competitive choices fortify operations, technology, processes, and organizational decision-making to work inclusively and achieve a sustainable market position (Zulfadli et al., 2019; Byun et al., 2020; Ding et al., 2020). The phenomenon of competitive choices has attracted many researchers (Periansya et al., 2018). Regularity in delivering services or goods is a fundamental practical component of competing firms (Kim and Chun, 2018). Competition is acquiring the same level of activities by two or more firms primarily based on quality, suppliers, raw material, and quantity (Tidström and Hagberg-Andersson, 2012). Usually, firm competition is based on market trends, technological advancements, micro and macro-economic factors, and social factors (Tidström and Hagberg-Andersson, 2012). In today’s cutthroat competitive scenario, every firm holds rivalry against each other, but several opportunities also exist to meet customer demands per their expectations (Arokiasamy, 2013). The five forces of competition drawn by Porter (1979) are the most used factors to address competition (Tallman et al., 2018). The core aim of Porter (1979) model is to study, provide, and identify each force that applies pressure on the competitors (Porter, 1980). Studies have argued that three approaches exist in competition, i.e., differentiation, focusing, and cost leadership (Porter, 1980, 1985; Zada et al., 2022b). Differentiation describes the ultimate characteristic that easily differentiates a firm’s offering from competitors. Cost leadership achieves a sustainable market position through effective cost minimization (Porter, 1980, 1985; Saeed et al., 2022). Employees carry out responsibilities to minimize competitors’ stresses (Jones et al., 2017; Gorsira et al., 2018). Globalization has brought the vast world market to a single place or platform, and competitive activities mostly rely on norms that affect an organization’s decisions (Luft, 2016). Managers overall choose various criteria for competition, i.e., a competitive edge would not be considered favorable operating in a different culture (Jones et al., 2017).

**FIGURE 1:**
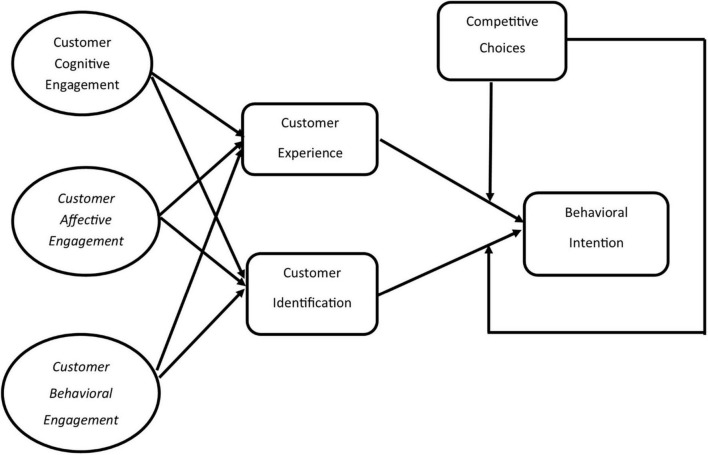
Research model of the study.

Customers have several competitive choices and opportunities to opt. Experience, interaction, knowledge, and know-how of the product can contribute toward the intention relating to a product, brand, service, or individual. Similarly, interaction with a product or brand can identify the product from a competitor, as shown in Figure 1.

### Hypothesis

From the literature review, the following working hypotheses emerged for this study.

H1: Customer effective engagement has a positive significant effect on customer experience.
H2: Customer effective engagement has a positive significant effect on customer information.
H3: Customer cognitive engagement has a positive significant effect on customer experience.
H4: Customer cognitive engagement has a positive significant effect on customer information.
H5: Customer behavioral engagement has a positive significant effect on customer experience.
H6: Customer behavioral engagement has a positive significant effect on customer information.
H7: Customer experience has a positive significant effect on behavioral intention.
H8: Competitive choices significantly moderate between customer experience and behavioral intention.
H9: Competitive choices significantly moderate between customer identification and behavioral intention.

## Materials and Methods

As organizations and customers sustain an informational revolution, new areas of marketing continue to emerge (Jain and Singh, 2002). One and a half-decade ago, the focus turned from treating customers as a matter engaged in the value creation of the business processes. The hypotheses mentioned earlier were explored using data obtained from Pakistan SMEs involved in the granite business. The area for research was chosen because more SMEs are based in the targeted area. Due to the lack of a proper database of SMEs in Pakistan, it was challenging to identify sampling techniques for this study. The author acquired the list of registered business units from Pakistan’s taxation network. The author asked the owners to take part in the survey because they are responsible authorities and aware of the administrative operations of their business. This study followed a convenience sampling technique. A questionnaire in complex form was applied instead of an email survey in a developing country with a lower response rate. For this research, a structured questionnaire was used to collect data because such methods are efficient in collecting data on the sample’s attitudes, opinions, and values. The author hired ten enumerators with minimum field experience of approximately 5 years from national organizations covering different cities to facilitate data collection. In total, 150 copies of the questionnaire were provided to each of them and asked to distribute these questionnaires to the owner, managers of various SMEs operating in the study field. The survey was conducted in English, which is the instruction medium. The questionnaire ensured that the data collected through this survey will be utilized only for the research purpose and will not be compromised at any cost. In addition, to encourage accurate and straightforward responses, the cover letter stressed that participation was voluntary, secret, and anonymous, and that was no true or false response for specific items. Out of 400 distributed questionnaires, 249 were retrieved, while only 216 were helpful for analysis with a response rate of 54%. Demographic figure proposed that the age of 93% (*n* = 216) respondents varied from 20 to 30 years while 77.3% were male.

The questionnaire was designed adopting the scales of previous authors and modified as per the requirement of the proposed study. Customer engagement comprised three multi-dimensions, i.e., cognitive, and behavioral engagement with three items equally, while affective engagement with four items. The scale was adopted from Hollebeek et al. (2014). Customer experience is a phenomenon primarily studied in strategic marketing that comprises 12 items adopted from Tsaur et al. (2007) and Brakus et al. (2009), respectively. Customer identification comprises four items adapted from Kumar and Kaushik (2018). The scale of behavioral intention included three items adapted from Coudounaris and Sthapit (2017). All the items were measured through a 5-point Likert scale. Finally, an open-ended item was adopted from the study of Khan (2014) for competitive choices adjusted at the end of the demographic section. The statistical analysis of demographics for the data were carried out in SPSS version 20 while correlation, regression, and CFA were carried out in AMOS version 16. Data were analyzed through various tests, i.e., frequency analysis, reliability, regression, moderated regression, and structural equational modeling, to check the consistency of the data.

## Results

The study included 77% of male and 23% of female respondents with 86% of single status along with 160 candidates ranging from the age of 20–25. Most of the respondents were graduates, respectively, who were easy to be gathering data. The reliability of the scales was measured through Cronbach’s alpha which is as follows:

Table 1 depicts that the scales adopted in the study are reliable although their liability for affective and behavioral engagement was less but still acceptable (Hepola et al., 2016). The relationship among the variables of the study was measured through correlation analysis which is as follows:

**TABLE 1:** Reliability analysis.

Scale	Items	Cronbach’sα
Customer engagement (Cognitive engagement)	3	0.795
Customer engagement (Affective engagement)	4	0.538
Customer engagement (Behavioral engagement)	3	0.510
Customer experience	12	0.899
Customer identification	4	0.756
Behavioral intention	3	0.662

Table 2 depicts the relationship among all the variables of the study. The table states that the relationship among all the variables of the study is positive and significant. The effect of the independent on dependent variable was analyzed through regression analysis which is as follows:

**TABLE 2:** Correlation analysis.

Variables	CECE	CEAE	CEBE	CE	CI	BI
CECE	1.000	–	–	–	–	–
CEAE	0.425**	1.000	–	–	–	–
CEBE	0.487**	0.573**	1.000	–	–	–
CE	0.690**	0.545**	0.629**	1.000	–	–
CI	0.532**	0.413**	0.451**	0.723**	1.000	–
BI	0.460**	0.384	0.321**	0.595**	0.615**	1.000

**Correlation is significant at the 0.01 level (2-tailed). (CECE, Customer Engagement Cognitive Engagement; CEAE, Customer Engagement Affective engagement; CEBE, Customer Engagement Behavioral Engagement; CE, Customer Experience; CI, Customer Identification; BI, Behavioral Intention).

Table 3 indicates the effect of independent on dependent variable study is analyzed. The total impact of cognitive engagement on customer experience is 61%, positively significant, respectively. Similarly, the actual effect of affective engagement on customer experience is 54% which is positively influential, respectively. In comparison, behavioral engagement has a total impact on customer experience is 62%, respectively. The cognitive engagement accumulates on customer identification measured 50%, respectively. An effective engagement affects customer identification calculated 44%, which is positively significant, respectively. Behavioral engagement has a total impact on customer identification estimated at 48%, which is positively substantial. Customer experience has a significant positive effect of 61% on behavioral intention. Similarly, customer identification has a significant positive impact of 58.6% on behavioral intention.

**TABLE 3:** Regression analysis.

Independent variable	Dependent variable	β	Std. error	Sig.
CECE	CE	0.606	0.043	0.001
AE	CE	0.541	0.057	0.001
BE	CE	0.622	0.053	0.001
CECE	CI	0.503	0.055	0.001
AE	CI	0.442	0.067	0.001
BE	CI	0.480	0.065	0.001
CE	BI	0.611	0.056	0.001
CI	BI	0.586	0.051	0.001

*CECE, Customer Engagement Cognitive Engagement; CEAE, Customer Engagement Affective Engagement; CEBE, Customer Engagement Behavioral Engagement; CE, Customer Experience; CI, Customer identification; BI, Behavioral Intention.*

Competitive choices use moderators between them, customer experience, and customer identification on behavioral intention. Therefore, moderated regression analysis was carried study the effect which is as follows:

Table 4 indicates the effect of moderation between the independent and dependent variables. The impact of independent on the dependent variable in the presence of competitive choice is minimal, i.e., 0.7% with the insignificance of 0.853, thus, proving positively insignificant. Similarly, the independent effect on the dependent variable in the presence of competitive choice is minimal, i.e., only 3.2% with the insignificance of 38%, thus, proving positively insignificant, respectively. Therefore, it is established from both the analysis that moderation has significantly less, or almost no effect in the study also analyzed the survey through confirmatory factor analysis, which was carried out in AMOS-16, which is as follows:

**TABLE 4:** Moderation regression analysis.

Independent variable	Moderator variable	Dependent variable	β	Sig.
CE	CC	BI	0.007	0.853
CI	CC	BI	0.032	0.378

**FIGURE 2:**
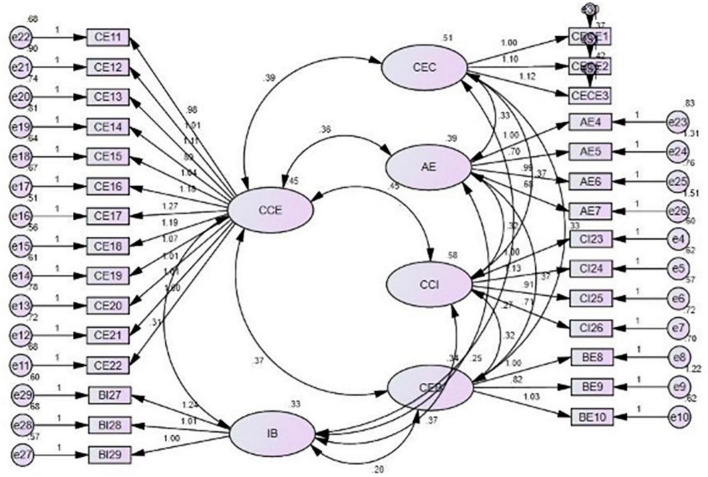
Confirmatory factor analysis.

Confirmatory factor analysis provides the author to better understand a concept that is measured by various predictors. Similarly, the study was carried out in the granite sector of Pakistan and the factors that influence the behavioral intention of customers, as shown in Figure 2 and Table 5.

**TABLE 5:** Confirmatory factor analysis.

Items	Standardized factor loadings	95% CI	*R* ^2^
CECE 1	0.069	[0.467, 0.771]	0.678
CECE 2	0.075	[0.447, 0.831]	0.792
CECE 3	0.064	[0.438, 0.749]	0.777
AE4	0.074	[0.562, 0.891]	0.563
AE5	0.078	[0.534, 0.904]	0.353
AE6	0.122	[1.013, 1.446]	0.576
AE7	0.072	[0.454, 0.812]	0.325
BE 8	0.068	[0.552, 0.825]	0.571
BE 9	0.072	[0.544, 0.944]	0.395
BE 10	0.078	[0.616, 0.975]	0.609
CE 11	0.062	[0.470, 0.774]	0.625
CE 12	0.059	[0.448, 0.707]	0.582
CE 13	0.055	[0.392, 0.648]	0.656
CE 14	0.069	[0.535, 0.870]	0.556
CE 15	0.065	[0.510,0.818]	0.659
CE 16	0.080	[0.664,0.997]	0.696
CE 17	0.075	[0.603, 0.911]	0.767
CE 18	0.090	[0.692, 1.167]	0.732
CE 19	0.069	[0.544, 0.866]	0.679
CE 20	0.094	[.613, 1.083]	0.610
CE 21	0.131	[1.101, 1.559]	0.626
CE 22	0.087	[0.591, 0.956]	0.634
CI23	0.150	[1.286, 1.763]	0.701
CI24	0.065	[0.399, 0.761]	0.737
CI25	0.076	[0.421, 0.913]	0.678
CI26	0.077	[0.446, 0.843]	0.540
BI27	0.068	[0.441, 0.799]	0.676
BI28	0.051	[0.267, 0.507]	0.573
BI29	0.056	[0.322, 0.523]	0.604

*Model fit indices: RMSEA = 0.065, p = 0.001, CFI = 0.869, TLI = 0.854, SRMR = 0.073.*

## Conclusion and Discussion

The study, which was carried out in a production sector of granite, which is considered one of the leading industries globally, consists of some practical implications. Quan and Wang (2004), Lemon and Verhoef (2016), and Le et al. (2019) suggested experiential industry as a crucial element of the economy to achieve a sustainable competitive edge, survival, and advantage. Therefore, developing an experiential environment by adopting the marketing perspective of experience is vital in the production sector of an economy. This study proposed a theoretical framework to examine the impact of the dimensions of customer engagement on customer experience on behavioral intention. However, production managers can create systems of experiential marketing like visits to factories, granite mines, and warehouses, and publicity programs to increase and facilitate entrepreneurship. Such programs should also be developed, increasing customer experience and identification, e.g., by providing customers with a handful of exposure to the granite industry. These programs will boost experience, loyalty, reliability, and self-identification. However, an increase in customer engagement can further develop customer experience, identification, and eternally behavioral intentions.

Extensive research has explored customer engagement with various technologies, particularly experiential marketing, i.e., gamification (Bryce et al., 2015; Ahn and Back, 2018). The study discovers customer engagement with the offerings of experiential marketing more widely, as in the proposed model of the study (see Table 6). Results thereby lead toward strategic value to production managers regarding customer experience design, which are in line with the proposed research findings, suggesting highlighting the exploration of the dimensions of customer engagement, i.e., affective, cognitive, and behavioral. For instance, production managers may achieve this goal by providing detailed and rich information, i.e., guiding the customers about the process of granite blocks extraction from rocks, the human resources involved, the process of slicing as per accurate machinery with proper measurement. This is a value-creation process that can quickly generate significant revenues in terms of existing and new customers (Rather, 2019; Zhang et al., 2020). To create or provide more intensive knowledge of the field, virtual reality techniques can give a closer glimpse of the production capacity from the inception till the installation of the rock in monuments and other architecture.

**TABLE 6:** Summary of analysis.

	Hypothesis	Result
H1	Customer affective engagement has a positive significant effect on customer experience.	Accepted
H2	Customer affective engagement has a positive significant effect on customer information	Accepted
H3	Customer cognitive engagement has a positive significant effect on customer experience	Accepted
H4	Customer cognitive engagement has a positive significant effect on customer information.	Accepted
H5	Customer behavioral engagement has a positive significant effect of customer experience.	Accepted
H6	Customer behavioral engagement has a positive significant effect of customer information	Accepted
H7	Customer behavioral engagement has a positive significant effect of customer information	Accepted
H8	Customer experience has a positive significant effect on behavioral intention	Accepted
H9	Competitive choices significantly moderate between customer experience and behavioral intention	Rejected
H10	Competitive choices significantly moderate between customer identification and behavioral intention.	Rejected

Experiential marketing experts provide various online and offline techniques. In a highly competitive production context full of customer demands, durability, reliability of services, social media platforms, online brand communities are the definite and perfect platforms to attract customers psychologically and customer identification and know-how of the industry (Harrigan et al., 2018a; Hollebeek et al., 2019). Developing online social platforms can encourage customer experience and further increase customer intellectual experience and information in the granite sector (Islam et al., 2019). Production marketers can also adopt customized services to enhance customer engagement and experience by utilizing massive data. Studying customers’ behavioral data can be crucial to developing after-sales services and promotional activities with advancement for new customers. For example, amazon.com, ebay.com, and Alibaba.com facilitate their customers by posting reviews and ratings of their experiences. Applying massive data may construct novel insights by discovering the relationship between marketing activities, customer experience, identification, and future intentions. However, it can adopt public marketing activities such as expos, campaigns, charity events, and sponsorship activities to grab the further attention of new customers (Bhattacharya and Sen, 2003; Rather and Hollebeek, 2019). The findings also recommend developing customer loyalty. Production sector managers can create a positive and unique production experience and identification compared with competitors. Production managers strive a lot to produce unique customer brand identification. However, a sustainable difference in offering in the services may be applied to identify distinctiveness in fulfillment and attending customer identification (Hultman et al., 2015; Rather, 2019) which can further enhance customer loyalty (Kumar and Kaushik, 2018). Similarly, creating a brand success story, customer experience stories on YouTube, and other social media services can help a new customer in decision making. Virtual reality tools can further spice up experiences (Ahn and Back, 2018; Harrigan et al., 2018a).

Practical implications can clear ambiguities between two markets/groups; hence, a two-sided mechanism can be chased by the marketing manager, i.e., one group focus on new customers while the other one on the existing customers to enhance further engagement, experience, identification, and behavioral intention in the production market. Both the markets and groups carry mutual benefits; therefore, production managers should develop product marketing plans, promotional plans, and strategies for both these segments. Brochures, photographs, and promotional videos demonstrate various features of various stones with enhanced communication on the social media platform. Cognitive customer engagement is considered vital in almost all the marketing practices of customer engagement for both new and existing customers, which can highlight the promotional activities. Similarly, governmental authorities should focus on customer engagement behavior, which is the second component of customer engagement. Similarly, brokers and retailers should focus on the effective behavior of customer engagement, i.e., attracting customers by keeping various samples of different shades, measurements, and thicknesses.

Furthermore, “ACT” technique is considered the most crucial element of customer experience, which is accompanied by sense, think, feel, and relate aspects for both existing and new customers, which should be a center of attention in the promotional activities. Existing customers have a high degree of identification and an increased behavioral intention rather than new customers. Thus, in product marketing, new customer production marketers offer effective and renowned production information to upgrade their experience and behavioral intentions (Liu et al., 2012). Correspondingly, for existing customers, production marketers can design marketing techniques to improve identification and desire to recommend their seller to other customers as well (Fakeye and Crompton, 1991; Wang, 2004).

Finally, this study consists of a few limitations. The study has relied on cross-sectional data, whereas future studies can explore the relationship between the variables (Hollebeek et al., 2019). The study can further be tested and analyzed especially in the hospitality industry. Whereas future studies can imitate the results from this study across all the sectors of various industries or countries as well. Future studies can further explore the role of “ACT” technique. Furthermore, the studies can be carried out in consumer psychology to better explore and study the dimensions of customer experience and customer engagement.

## Data Availability Statement

The raw data supporting the conclusions of this article will be made available by the authors, without undue reservation.

## Ethics Statement

The University Review committee (U.R.C.) involving Human Subjects for School of Business, Qingdao University, China, has reviewed the proposal stated above and confirmed that all procedures performed in studies involving human participants were in accordance with the ethical standards of the institutional research committee and with the 1964 Helsinki declaration and its later amendments or comparable ethical standards. The participants of this study was the SMEs involved in the granite business, Pakistan. Informed consent has been obtained from all subjects involved in this study to publish this paper. Further, formal approval was obtained from the competent authorities of the organizations that participated in the study. The university research committee approved all the procedures on research involving Human Subjects of Qingdao University, China.

## Author Contributions

BA, LZ, and SZ: conceptualization and writing—original draft preparation. BA, LZ, SZ, and SS: methodology. BA and LZ: software. LZ, SZ, and AV-M: validation. LZ, SS, and NC-B: formal analysis. SZ and SS: resources. GS-S, NC-B, and AV-M: writing—review and editing and funding acquisition. LZ and AV-M: supervision. BA and SZ: project administration. All authors have read and agreed to the published version of the manuscript.

## Conflict of Interest

The authors declare that the research was conducted in the absence of any commercial or financial relationships that could be construed as a potential conflict of interest.

## Publisher’s Note

All claims expressed in this article are solely those of the authors and do not necessarily represent those of their affiliated organizations, or those of the publisher, the editors and the reviewers. Any product that may be evaluated in this article, or claim that may be made by its manufacturer, is not guaranteed or endorsed by the publisher.

## Appendix

**Table:**

S#	Questions	Scale				
	Customer engagement (cognitive engagement)					
1	Dealing with my specific seller gets me to think about our business relationship.	5	4	3	2	1
2	I think about this seller a lot when I’m dealing.	5	4	3	2	1
3	Dealing with this specific industry stimulates my interest to learn more about it.	5	4	3	2	1
	**Customer engagement (affective engagement)**					
4	I feel very positive when I deal with this seller.	5	4	3	2	1
5	Dealing with my specific seller makes me happy.	5	4	3	2	1
6	I feel good when I use the product of my specific seller.	5	4	3	2	1
7	I feel proud dealing with my specific seller.	5	4	3	2	1
	**Customer Engagement (Behavioral Engagement)**					
8	I spend a lot of time dealing with my specific seller compared to other sellers.	5	4	3	2	1
9	Whenever I desire to buy this product, I usually deal with my specific seller.	5	4	3	2	1
10	I deal with my specific seller the most.	5	4	3	2	1
	**Customer experience**					
11	My specific seller always has something interesting.	5	4	3	2	1
12	My specific seller tries to engage my senses.	5	4	3	2	1
13	My specific seller lacks sensory appeal for me.	5	4	3	2	1
14	My specific seller makes me respond in an emotional manner.	5	4	3	2	1
15	My specific seller tries to put me in a certain mood.	5	4	3	2	1
16	My specific seller stimulates my curiosity.	5	4	3	2	1
17	My specific seller tries to intrigue me.	5	4	3	2	1
18	My specific seller product tries to appeal to my creative thinking.	5	4	3	2	1
19	I would like to share what I experienced with my specific seller.	5	4	3	2	1
20	I would like to take pictures of my specific seller products.	5	4	3	2	1
21	My specific seller induces me a sense of identity toward ecological conservation.	5	4	3	2	1
22	I would buy some souvenirs which are related to my seller’s destination.	5	4	3	2	1
	**Customer identification**					
23	When someone criticizes my specific seller, it feels like a personal insult.	5	4	3	2	1
24	I am very interested in what others think about this my specific seller.	5	4	3	2	1
25	If someone criticizes my specific seller, I feel embarrassed.	5	4	3	2	1
26	When someone praises my specific seller, it feels like a personal compliment.	5	4	3	2	1
	**Behavioral intention**					
27	I plan to do business with my specific seller again in the near future.	5	4	3	2	1
28	I would recommend my specific seller to my friends or other business associates.	5	4	3	2	1
29	I plan to participate in the same activities my specific seller is involved in.	5	4	3	2	1
						

**Demographics**

(**Note:** Please encircle that best suit you)

**Name (Optional): Gender:** Male Female

**Marital Status:** Single Married Divorced

Separated

**Are You:** Employed Unemployed

**Age:** 20–25 26–30 31–35 36–40 40+

**Education:** Metric Intermediate Bachelors Masters MS/MPhil Doctorate

**Organization (Optional):**

**Phone/Mobile/E-mail (Optional):**

**How many same levels of sellers are available in this area?**
